# Convergent Geroscience: Integrating Social, Population, and Biological Aging Research to Healthy Aging

**DOI:** 10.1111/acel.70801

**Published:** 2026-11-13

**Authors:** Naibedya Dutta, Eleanor Hayes‐Larson, Victor A. Ansere, Gilberto Garcia, Eileen M. Crimmins, Sean P. Curran, Bérénice A. Benayoun, Jennifer A. Ailshire, Ryo Higuchi‐Sanabria

**Affiliations:** ^1^ Leonard Davis School of Gerontology University of Southern California Los Angeles California USA

## Abstract

Aging research has advanced along two largely parallel paths. Experimental biology has transformed aging research by establishing that aging is biologically malleable and identifying conserved molecular and cellular mechanisms that regulate lifespan and healthspan. In parallel, population research has revealed substantial heterogeneity in human aging and identified social, environmental, behavioral, and genetic factors associated with differences in aging trajectories. Despite a shared goal of extending healthy aging, limited integration between these fields constrains progress. We argue that building upon these complementary advances and increasing greater integration between experimental biology and population approaches, termed *convergent geroscience*, can further accelerate the field. We highlight examples of this integration, including biological aging clocks that provide measurable readouts associated with social exposures and molecular processes, population‐informed model systems that incorporate heterogeneity, and population‐derived findings that catalyze mechanistic discovery. We further discuss how incorporating variability, context, and real‐world complexity into experimental design can improve translational relevance. Finally, we propose priorities for advancing this integrative framework. Convergent geroscience offers a path toward more effective, equitable interventions that can extend healthspan at scale.

## Graphical abstract

Convergent geroscience integrates the complementary strengths of experimental biology and population research. Experimental biology provides the mechanistic “bricks” of aging, while population research provides the “concrete” connecting these mechanisms across human heterogeneity. Together, they create a bidirectional framework for translating mechanistic discoveries into interventions that promote healthy aging across populations.

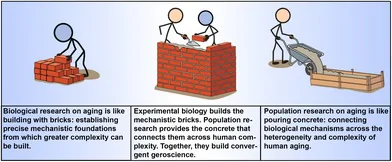

## 1 Introduction

Aging research has been transformed by experimental biology. Studies across yeast, *Caenorhabditis elegans* , *Drosophila*, mice, and mammalian cell systems established that aging is not simply an inevitable accumulation of damage but a biologically regulated and experimentally malleable process. Decades of mechanistic research have identified conserved pathways controlling longevity and age‐associated decline and ultimately gave rise to conceptual frameworks such as the “Biological Hallmarks of Aging,” encompassing genomic instability, cellular senescence, altered proteostasis, mitochondrial dysfunction, and other processes that contribute to aging pathology across species (López‐Otín et al. 2023). These discoveries provided the mechanistic foundation for modern geroscience and enabled development of interventions designed to directly modify biological processes of aging.

Population research has generated a complementary body of knowledge by defining how aging trajectories vary across individuals and populations and identifying social, environmental, behavioral, and genetic factors associated with that variation. Population researchers have proposed an analogous set of “Social Hallmarks of Aging,” encompassing interrelated social conditions that pattern health, aging, and longevity across the life course (Crimmins 2020). Yet these two highly successful research fields still interact less frequently than their shared goal of extending healthy aging would warrant. We propose that the next phase of geroscience can build upon the mechanistic foundation established by experimental biology by more systematically incorporating the heterogeneity, exposures, and aging trajectories revealed through population research. Conversely, experimentally tractable systems provide the essential means to move population‐level associations toward biological mechanism and causality.

The opportunity for integration emerges precisely because these approaches provide different forms of information, both with equal value. Experimental systems offer extraordinary control over genetics, environment, timing, and tissue specificity, allowing rigorous tests of causality that are generally impossible in human populations. Population studies, in turn, capture dimensions of human heterogeneity and life‐course exposure that cannot be fully reproduced experimentally. Connecting these strengths creates an opportunity to ask not only whether a biological mechanism regulates aging, but how strongly it contributes to aging across heterogeneous human contexts, and conversely, how population‐level observations can reveal new biological questions for mechanistic investigation.

To address this gap, the Geroscience Los Angeles Meeting (GLAM), an annual conference hosted by the Leonard Davis School of Gerontology at the University of Southern California and partnering institutions, University of California Los Angeles, and Cedars‐Sinai Medical Institute, aims to build a shared science platform that spans the breadth from molecules and cells to populations and societies. This integrative meeting promotes biological research that incorporates population relevance into model systems and population research that anchors its frameworks in biological mechanisms that can inform interventions. GLAM provides a forum for the concept of “Convergent Geroscience”, which aims to strengthen causal inference and broaden the translational relevance of aging research across diverse populations.

In this review, we first lay out the gap between experimental biology and population health research and its consequences, argue that integration across the two areas can benefit both, and lay out several examples of the potential for integration to benefit aging research. We conclude with a discussion of GLAM and similar scientific meetings as an exemplar of strategic activity for integration, as well as other recommendations to pave the way forward. Throughout, we refer to “experimental biology” as a broad term encompassing laboratory‐based investigations of biological mechanisms, including studies in model organisms, cell culture systems, and controlled experimental settings designed to test causal relationships. We use the term “population research” broadly to encompass large‐scale studies in human populations, including a variety of demographic, epidemiologic, sociologic, and other (e.g., genetic) research. While domains within population research are often conceptually distinct (e.g., research on social determinants of health versus genome‐wide association studies [GWAS]), they are often treated as part of a shared “non‐molecular” research space by experimental biologists and jointly offer critical entry points for linking human variation to biological mechanisms. Here, we use ‘population‐level heterogeneity’ broadly to encompass genetic, environmental, behavioral, and social sources of variation that shape aging trajectories across individuals and populations.

## 2 Experimental Biology and Population Research: Complementary Foundations for Geroscience

Funding for aging research, including from the National Institute on Aging (NIA), has grown substantially and consistently in recent decades (Kelley et al. 2024). Consistent with increased funding, publications related to aging research have also dramatically increased (Zhang et al. 2024). The rapid expansion of aging research has been driven by major advances across both experimental biology and population sciences. Experimental biology has progressed from identifying conserved regulators of lifespan to developing mechanistically informed interventions targeting fundamental aging processes, while population research has built increasingly rich longitudinal datasets that characterize how aging unfolds across diverse human lives and environments. These parallel advances have created an opportunity to move beyond simply describing mechanisms or population‐level variation toward determining how established aging mechanisms operate across heterogeneous human contexts and how observations from those populations can, in turn, generate new mechanistic questions.

Experimental biology produced several foundational discoveries that established modern geroscience. Genetic studies demonstrating that manipulation of single pathways could substantially alter lifespan fundamentally changed the view of aging, demonstrating that the aging process itself could be experimentally modified. Subsequent work identified conserved regulators of nutrient sensing, proteostasis, mitochondrial function, cellular senescence, stem cell maintenance, genome stability, and stress responses across species. Collectively, these discoveries shifted the field from simply describing age‐associated decline toward identifying causal mechanisms to target for therapeutic intervention (Kroemer et al. 2025). For example, interventions such as the mTOR inhibitor rapamycin (Harrison et al. 2009; Mannick et al. 2014), senolytic therapies like dasatinib and quercetin that selectively eliminate senescent cells (Justice et al. 2019; Zhu et al. 2015; Novais et al. 2021), and partial cellular reprogramming using the Yamanaka factors (OSKM: OCT4, SOX2, KLF4, c‐MYC) to rejuvenate exhausted stem cells (Ocampo et al. 2016; Takahashi et al. 2007), represent a growing pipeline of mechanistically informed gerotherapeutics.

The translation of these approaches into humans has also provided important insights into the challenges of moving from mechanistic discoveries in animal models to interventions for heterogeneous human populations. For example, clinical studies of mTOR inhibition have highlighted the importance of dose, treatment duration, pharmacokinetics, and tissue‐specific effects (Hartford et al. 2009; Choo et al. 2013; Wang et al. 2024; Roark and Iffland 2025), while studies of senolytic therapies have underscored heterogeneity in senescent cell burden, target‐cell accessibility, and potential off‐target effects (Puri and Wagner 2023; Wuputra et al. 2020). Similarly, partial cellular reprogramming has demonstrated the potential to reverse age‐associated molecular and cellular phenotypes, while also revealing substantial challenges related to delivery, control of reprogramming, and tumorigenic risk (Yücel and Gladyshev 2024; Simpson et al. 2021; Puri and Wagner 2023; Wuputra et al. 2020). These findings do not diminish the value of the experimental systems in which these interventions were discovered; rather, they illustrate how mechanistic discoveries establish new therapeutic possibilities while subsequent studies identify the biological and translational parameters that allow for their translational effectiveness in humans. Convergent geroscience can build on this foundation by incorporating information about human heterogeneity, exposure history, comorbidities, and population context to help determine how and in whom these interventions are most likely to be effective.

A major strength of experimental biology research has been the deliberate simplification of biological systems to enable rigorous mechanistic discovery. Animal and cellular studies frequently use controlled, low‐variability environments and isogenic or nearly identical genetic backgrounds to minimize biological noise (Kenyon 2010; Harper 2008). This reductionist approach is not a limitation of experimental biology, but a necessity: establishing the fundamental mechanisms that regulate aging requires controlling sources of variability sufficiently to identify, manipulate, and validate individual pathways. Building on our analogy presented in our graphical abstract, experimental biology first needed to “make the bricks” by defining the molecular and cellular mechanisms from which a broader understanding of aging could ultimately be constructed. Having now identified many of these foundational mechanisms, the field is increasingly positioned to ask how they operate within more complex and heterogeneous settings. Studies using genetically diverse populations, including outbred mouse cohorts (Harrison et al. 2009; Saul et al. 2019; Churchill et al. 2012) and wild isolates (Andersen and Rockman 2022; Rodriguez et al. 2012) have begun this transition. Beyond genetics, controlled laboratory environments also cannot fully reproduce the cumulative lifetime exposures that shape human aging, including social and environmental factors. Even beyond controllable factors, it is impossible to experimentally eliminate the stochastic components of aging arising from random, non‐genetically encoded processes such as damage accumulation, transcriptional noise, and epigenetic drift (Hruby et al. 2025). Convergent geroscience therefore represents a natural bridge rather than a replacement for reductionist biology by building upon the mechanistic “bricks” established through decades of controlled experimentation and systematically introducing genetic, environmental, social, and stochastic heterogeneity to determine how these mechanisms operate across the complexity of real‐world, human aging.

In parallel with advances in experimental biology, population research fields, including epidemiology, demography, sociology, and others, have also made profound contributions to understanding how aging occurs throughout the life course. For example, large‐scale longitudinal and cross‐sectional studies have consistently demonstrated that social and structural factors including education, income, occupational exposure, neighborhood environment, race‐related disadvantage, and early‐life adversities are strong predictors of lifespan and healthspan at later ages (Crimmins 2020; Crimmins 2015). Landmark population‐based longitudinal studies including the Health and Retirement Study (HRS) (Sonnega et al. 2014), Dunedin Multidisciplinary Health and Development Study (Poulton et al. 2015), observational extensions of the Women's Health Initiative (WHI) (Langer et al. 2003), and the Normative Aging Study (NAS) (Bell et al. 1972), have shown that individuals exposed to chronic socioeconomic disadvantages or cumulative stress experience earlier onset of multimorbidity, accelerated functional decline, and reduced life expectancy. Importantly, large longitudinal cohorts also enable identification of individuals who deviate from expected trajectories, such as those who age well despite high‐risk social exposures (Glonti et al. 2015). These “outlier” cases can provide powerful starting points for mechanistic biological investigation, allowing population findings to guide experimental discovery.

More recently, the incorporation of biological biomarkers of aging, such as epigenetic clocks (DNA methylation‐based predictors of biological age or health outcomes, explained in more detail below), inflammatory markers, and composite physiological indices into population‐based cohorts has begun to link social exposures to measurable biological processes, providing evidence that social conditions may shape the rate of biological aging itself. These findings have been instrumental in shifting aging research away from a purely disease‐focused framework toward a life course model, proposing that aging is shaped by social determinants across the life course, often well before clinical pathology emerges.

Despite their explanatory power at the population level, many discoveries in population research, particularly on social drivers of aging, face important limitations complementary to those of biological aging research. For example, population studies rely on observational designs that are vulnerable to residual confounding, which can limit causal inference and complicate translation into therapeutic strategies (Ioannidis 2005; Hernán and Robins 2016; VanderWeele 2016). Even after identifying social exposures that causally affect accelerated aging and predict who may be at the highest risk, population research often lacks the mechanistic resolution needed to explain how social experiences translate into biological outcomes. For example, associations of low socioeconomic status, discrimination, or early‐life trauma with adverse aging outcomes are robust and reproducible (Lim et al. 2022; Braveman et al. 2011), but how these link to biological phenotypes such as immune dysfunction, metabolic dysregulation, or neurodegeneration is not well defined (Cuevas et al. 2020; Adler and Rehkopf 2008). The next step, which includes identification of actionable molecular targets for therapeutic interventions, requires significant additional biological work.

Together, these traditions have created complementary foundations for modern geroscience. Experimental biology has established causal mechanisms and demonstrated that biological aging can be manipulated, while population research has mapped the substantial heterogeneity of aging across human lives and environments. Convergent geroscience seeks to build upon these successes by creating a more iterative relationship between the two. Population observations can identify contexts, exposures, and phenotypes requiring mechanistic explanation, while experimental biology can establish causality and identify actionable pathways that can subsequently be evaluated across human populations.

## 3 The Case for Integration

The case for convergent geroscience does not rest on a limitation of either experimental biology or population research, but on the opportunity created by their individual success. Once causal mechanisms of aging have been identified, a critical next question is how those mechanisms operate within the genetic, environmental, behavioral, and social heterogeneity of human populations. Likewise, population‐level associations become substantially more actionable when experimental approaches can identify the molecular and cellular mechanisms through which they influence aging.

A growing body of population research, including demographic and epidemiologic studies, shows that social exposures are not simply peripheral factors for aging but act as direct and fundamental determinants of health over the human life course. Early demographic studies indicate that inherited genetic variation may explain only a modest share of human lifespan variability. For example, a meta‐analysis of twin and sibling studies estimated that hereditary factors only explain approximately 12% of lifespan variability in siblings and 25% in twins (Herskind et al. 1996), and may be as low as 7% after correction for assortative mating (i.e., the tendency to choose reproductive partners who are similar in important social traits like income, education, personality, or lifestyle) (Ruby et al. 2018). However, estimates of the genetic contribution to human lifespan are highly sensitive to how lifespan and sources of mortality are modeled. For example, a recent study found that inclusion of extrinsic causes of death, such as accidents and infections, can obscure the underlying genetic contributions to age‐related mortality. In fact, after mathematically accounting for extrinsic mortality and analyzing multiple twin and sibling cohorts, the heritability of intrinsic human lifespan can exceed 50% (Shenhar et al. 2026). One important consideration is that separating intrinsic from extrinsic mortality is itself complex, as susceptibility to ostensibly extrinsic causes of death may also reflect interactions among genetic, behavioral, environmental, and social factors.

At the level of individual genetic variants, comprehensive genome‐wide association studies (GWAS) find very small genetic contributions to longevity compared to environmental or social factors (Deelen et al. 2019). In contrast, social factors, including lower education, lower socioeconomic status, racially patterned disadvantage, and cumulative adversity, produce dramatically larger differences in life expectancy and healthspan across populations (Crimmins 2020). Thus, rather than establishing a single fixed genetic contribution to longevity, ongoing research shows that measurements of lifespan emerge from the interplay between inherited biology and the environmental and population contexts in which aging occurs.

Concurrently, it is clear that exposure to social drivers of aging is associated with measurable biological consequences across a variety of mechanistic pathways (Figure 1). Large cohort studies including the HRS (Sonnega et al. 2014; Juster and Suzman 1995), the Dunedin Study (Poulton et al. 2015), WHI (Langer et al. 2003), and the NAS (Bell et al. 1972) have all revealed that older adults with histories of low SES, childhood adversity, or chronic discrimination show accelerated epigenetic age, a DNA methylation‐based estimate of divergence between chronological age and biological age (Kusters and Horvath 2025). Early‐life poverty predicts older epigenetic age at midlife (Levine et al. 2015), while individuals experiencing chronic racial discrimination show significantly accelerated aging associated with a 26%–43% higher risk of all‐cause mortality (Levine and Crimmins 2014). Similarly, children exposed to abuse or an unstable home exhibited shorter telomeres in blood cells and accelerated epigenetic aging as early as 10 years of age (Shalev et al. 2013), demonstrating that aging pathways can be biologically programmed early in life. Social adversity also induces chronic inflammation, another biological hallmark of aging often referred to as “inflammaging”. Adults living in socioeconomically disadvantaged neighborhoods exhibit chronically elevated circulating C‐reactive protein (CRP) and interleukin‐6 (IL‐6) levels (Petersen et al. 2008; Muscatell et al. 2020; Gimeno et al. 2007), common measures used to characterize inflammaging (Ferrucci and Fabbri 2018). Other known biological consequences of social factors such as low SES or racial marginalization include increased glucocorticoid resistance, heightened NF‐κB transcriptional activity, and accelerated immunosenescence, including reduced naïve T cell pools and impaired vaccine responses (Miller et al. 2009). Ultimately, these studies support the idea that social forces are associated with measurable biological manifestations of aging.

**FIGURE 1:**
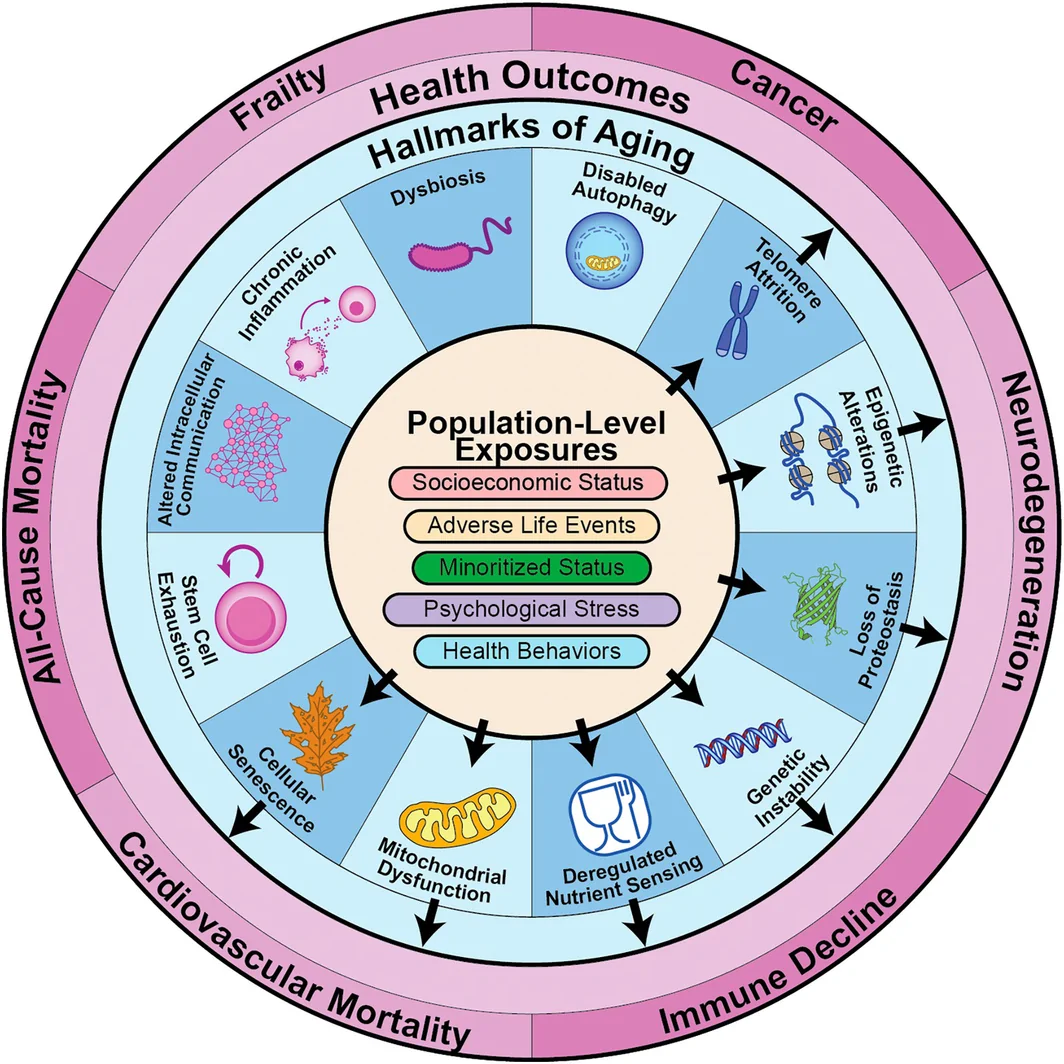
Population‐level exposures are linked to biological hallmarks of aging and downstream health outcomes. This schematic illustrates selected relationships between population‐level exposures, biological hallmarks of aging, and aging‐related health outcomes discussed in this Review. Arrows extending from population‐level exposures to individual hallmarks indicate relationships for which published studies have associated social, environmental, behavioral, or other population‐level exposures with the indicated biological aging process. Arrows extending outward from individual hallmarks represent which of these processes linked between population‐level exposure and biological aging have been implicated in downstream aging‐related health outcomes, including frailty, cancer, neurodegeneration, immune decline, cardiovascular mortality, and all‐cause mortality. Together, these relationships illustrate how population‐level exposures may become biologically embedded through established aging processes and ultimately contribute to differences in health and disease across populations. The relationships shown are intentionally selective and reflect examples discussed in this review rather than an exhaustive mapping of known associations. Arrow placement around the outer ring does not indicate a specific relationship with the adjacent health outcome; rather, the outer arrows collectively indicate progression from population‐level exposure to biological aging processes toward downstream health outcomes. Similarly, the different shades of blue used for biological hallmarks and purple used for health outcomes are included solely for visual distinction and readability and have no biological or quantitative significance.

The critical role of psychosocial factors on biological lifespan and healthspan is visible even in animal models. For example, conventional laboratory housing for rodents can be highly stressful for animals as cage sizes are kept at a minimum to reduce costs, and often contain little more than food, water, and some form of bedding (e.g., corncob), a stark contrast to wild habitats where rodents can dig, burrow, and create nests that provide feelings of security and safety (MacLellan et al. 2021). A meta‐analysis found that conventional laboratory housing increased stress‐related diseases and mortality in mice and rats compared to “enriched” housing, defined as cages containing non‐standard resources such as running wheels or opportunities for other activities including climbing, gnawing, and exploration (Cait et al. 2022). Other experimental work demonstrates that rodents actively seek out environmental stimuli: in a foundational behavioral study, mice were shown to repeatedly “work” to gain access to resources, such as additional space, social contact with other mice, climbing structures, and running wheels. Mice were willing to “pay” effort in the form of traversing a shallow pool of water to access these non‐food components, suggesting that opportunities for voluntary social enrichment are perceived as highly valuable to mice (Sherwin 1996). Together, these findings indicate that psychosocial conditions in laboratory settings are not merely controlled experimental context, but modifiable determinants of stress physiology and disease risk, and in turn, lifespan and healthspan.

Consistent with these findings, lifelong social isolation reduced lifespan in multiple animal models, including carpenter ants (Koto et al. 2023) and rats (Menich and Baron 1984), and phylogenetic comparative analysis shows that group‐living species (i.e., social animals) generally live longer than solitary species (Zhu, Liu, et al. 2023). Moreover, chronic psychosocial stress also reduced lifespan in C57BL/6J mice, one of the most common mouse models used in aging biology. Specifically, when animals were stratified based on social status, subordinate mice defined as those that had higher frequency of receiving aggression or attack, and lower frequency of initiating attack, had lower access to resources, and exhibited more defensive behaviors, showed significantly higher corticosterone levels, elevated inflammatory cytokines, increased senescent burden, early signs of cardiovascular pathology, and reduced median and maximal lifespan compared to their more aggressive counterparts (Razzoli et al. 2018). Importantly, these studies illustrate a growing experimental biology literature that directly tests how social conditions influence aging‐related biology. Beyond the mouse studies described above, experimental manipulation of social status in rhesus macaques demonstrated that social rank can causally alter immune cell composition and gene regulation, including inflammatory and antiviral response pathways, with subsequent work identifying corresponding changes in chromatin accessibility and glucocorticoid‐responsive gene regulation (Snyder‐Mackler et al. 2019). Taken together, these data support the idea that social environment is an important modifier of aging biology rather than merely a peripheral contextual variable. Importantly, recognition that social and environmental exposures influence aging biology does not imply that downstream gerotherapeutic interventions will be ineffective. Rather, convergent geroscience suggests that sustained upstream exposures may continuously reinforce biological damage accumulation, potentially limiting durability or accessibility of interventions if broader determinants of health are not also addressed. In parallel, interventions targeting upstream social and environmental factors may exert pleiotropic benefits across multiple biological aging pathways simultaneously, potentially complementing gerotherapeutic approaches focused on individual mechanisms.

Environmental conditions, especially those patterned by socioeconomic inequality, are increasingly recognized as important contributors to population aging with biological relevance. Factors like pollution, noise, and toxicant exposure disproportionately affect low‐income and racially marginalized communities and serve as additional accelerators of aging (Casey et al. 2017; Hajat et al. 2015; Clark et al. 2014). For example, global ambient air pollution, such as fine particle matter, is estimated to reduce life expectancy by ~2.9 years worldwide, with U.S. studies showing that long‐term fine particle matter exposure shortens life expectancy by ~0.5–0.6 years, even at exposures under current regulatory limits (Lelieveld et al. 2020; Bennett et al. 2019). Experimental biology studies provide mechanistic context to these population‐level patterns: exposure to environmental toxicants accelerate core biological hallmarks of aging across diverse organisms. In rodents, chronic exposure to particulate air pollution induces systemic inflammation (Cory‐Slechta et al. 2023), mitochondrial dysfunction (Li et al. 2015), vascular injury, and cognitive decline (Shou et al. 2020). Perhaps most strikingly, inhalation of airborne particulate matter in mice resulted in increased Alzheimer's Disease (AD) pathology, including microglial activation, blood–brain barrier disruption, and increased amyloid‐β aggregation (Liu et al. 2017; Chen et al. 2022; Finch and Morgan 2020). Similar aging‐promoting effects have been observed in invertebrate model systems: exposure of *C. elegans* to cigarette smoke resulted in a significant reduction in ability to clear pathogenic bacteria (Green et al. 2009), and exposure to traffic‐related air pollutants significantly reduced lifespan (Chung et al. 2020). In *Drosophila*, exposure to particle matter induced inflammatory response, increased oxidative stress, and reduced longevity by up to 50% (Wang et al. 2017). Across numerous species, environmental pollutants similarly activate conserved inflammatory responses, oxidative stress response pathways, and negatively impact protein homeostasis and neuronal health (Olloquequi et al. 2024; Wang et al. 2021), reinforcing that environmental disadvantage is increasingly recognized as an important modifier of biological aging trajectories. Conversely, exposure to environmentally enriching settings appears to buffer aging processes, even in animal models (Lockworth et al. 2015; Veness et al. 2025; Yamashita et al. 2018). These findings highlight that environmental exposures function not merely as background context, but as modifiable exposures that influence aging trajectories across species.

Together, these studies illustrate the complementary power of experimental biology and population health approaches. Population studies identify social and environmental exposures associated with divergent aging trajectories, while experimental systems can directly manipulate these exposures to establish causality and define the biological pathways through which they act. Convergent geroscience can make this exchange increasingly bidirectional, using observations from human populations to generate experimentally tractable hypotheses and mechanistic discoveries to refine interpretation of population‐level associations.

## 4 Proof‐of‐Concept and Emerging Examples

### 4.1 Biological Aging Clocks: A Convergent Framework Linking Social Exposure and Molecular Aging

Biological aging clocks represent one of the clearest examples of how population‐level observations can be linked to quantifiable biomarkers of biological aging. Biological aging clocks are measures that estimate the divergence between chronological and biological age using molecular features such as DNA methylation, creating a measurable link between lived experiences and underlying biology (Pal and Tyler 2016; Min et al. 2024). In this way, they provide a framework for studying how population‐level exposures, such as socioeconomic conditions, stress, and adversity, are associated with biological aging processes, which in turn can inform risk stratification and intervention strategies in human populations.

Successive generations of epigenetic clocks have expanded from predicting chronological age to estimating disease and mortality risk and, more recently, the pace of physiological aging (Table 1). First‐generation clocks, including Hannum and Horvath, were trained to predict chronological age, whereas later approaches such as PhenoAge and GrimAge incorporate health‐ and mortality‐related information, and DunedinPACE estimates the rate of physiological decline based on numerous health indicators (Li et al. 2022; Wang et al. 2022; Levine et al. 2018; Lu et al. 2019; Belsky et al. 2020; Belsky et al. 2022). These advances have made aging clocks increasingly useful for population research by providing quantitative biological measures that can be examined in relation to exposures, health trajectories, and interventions. The concept of biological aging clocks has also expanded beyond DNA methylation to include proteomic, metabolomic, transcriptomic, immune, neuroimaging, microbiome, and other molecular or physiological measures (Tanaka et al. 2018; Lehallier et al. 2020; Kuo et al. 2024; Mutz et al. 2024; Ibáñez De Opakua et al. 2025; Pilley et al. 2025; Franke and Gaser 2019; Sayed et al. 2021; Zhu, Chen, et al. 2023; Galkin et al. 2020; Wilmanski et al. 2021; Esparza et al. 2026; Ji et al. 2024; Ahadi et al. 2020).

**TABLE 1:** Overview of popular biological aging clocks: generations, modalities, and conceptual advances.

Clock generation/type	Representative models	Primary input/Data type	What it measures	Key conceptual advance	Strengths	Limitations	References
First‐Generation Epigenetic Clocks	Hannum Clock; Horvath Clock	DNA methylation (CpG sites)	Chronological age	First quantitative link between molecular features and age	High accuracy in predicting chronological age; multi‐tissue applicability (Horvath)	Weak association with functional decline, morbidity, or mortality	Hannum et al. (2013); Horvath (2013)
Second‐generation epigenetic clocks	PhenoAge; GrimAge	DNA methylation + clinical biomarkers (phenoage); methylation surrogates for proteins and smoking (GrimAge)	Biological age and disease risk	Shift from age prediction to health risk estimation	Better prediction of morbidity and mortality; integrates systemic health signals	Still largely statistical; limited mechanistic interpretability	Levine et al. (2018); Lu et al. (2019)
Third‐generation epigenetic clocks	DunedinPoAm; DunedinPACE	Longitudinal physiological data translated to methylation biomarkers	Pace of aging (rate of decline)	Measures dynamic aging rate instead of static age	Captures real‐time aging trajectory; sensitive to physiological decline	Requires complex longitudinal training data; still indirect mechanistic links	Belsky et al. (2020); Belsky et al. (2022)
Proteomic clocks	Various plasma protein‐based models	Circulating proteins	Functional physiological state	Adds functional, pathway‐level insight	Responsive to short‐term physiological changes; interpretable	Influenced by transient conditions; variability across individuals	Tanaka et al. (2018); Lehallier et al. (2020); Kuo et al. (2024)
Metabolomic clocks	(metabolite‐based models)	Small‐molecule metabolites	Metabolic state, mitochondrial function, inflammation	Captures near real‐time physiology	Reflects oxidative stress, lipid metabolism, systemic inflammation	Sensitive to diet, environment, and acute changes	Mutz et al. (2024); Ibáñez De Opakua et al. (2025)
Transcriptomic/single‐cell clocks	Single‐cell aging clocks (e.g., ribosomal activity signatures)	Gene expression (bulk or single‐cell RNA)	Cellular activity & aging trajectories	High‐resolution, cell‐type–specific aging	Identifies mechanisms (e.g., translation, proteostasis)	Technically complex; high variability	Zhu, Chen, et al. (2023), Zhu, Liu, et al. (2023)
Neuroimaging clocks (BrainAge)	MRI/PET‐based BrainAge models	Brain imaging data	Structural and functional brain aging	Organ‐specific aging measurement	Direct relevance to neurodegeneration	Expensive; limited accessibility	Franke and Gaser (2019)
Immune/inflammatory clocks	Cytokine and immune profiling models	Immune cell distributions, cytokines	Immunosenescence and inflammaging	Links aging with immune system dynamics	Captures chronic inflammation and stress biology	Highly context‐dependent; fluctuates with infections	Sayed et al. (2021)
Microbiome‐based clocks	Gut microbiome age models	Microbial composition & function	Host–microbiome aging interactions	Integrates environmental and lifestyle inputs	Captures host‐environment interactions; Provides insight into gut‐related health and systemic aging processes	High interindividual variability; Strongly influenced by short‐term factors such as antibiotics or illness	Galkin et al. (2020); Wilmanski et al. (2021)

Recent advances in transcriptomic clocks further suggest that some molecular signatures of aging are conserved across species. For example, a recent study integrating more than 11,000 transcriptomes across multiple tissues from mice, rats, macaques, and humans developed cross‐species transcriptomic clocks, highlighting conserved features of mammalian aging (Tyshkovskiy et al. 2026). Molecular aging measures may also provide useful tools for evaluating whether candidate interventions modify biological aging. Multi‐omic analyses in male monkeys, for example, showed that long‐term metformin treatment attenuated age‐associated molecular changes across multiple tissues (Yang et al. 2024). Extending this concept to humans, a recent phase 2a clinical trial applied six proteomic aging clocks to assess the effects of rentosertib, an anti‐fibrotic drug, and found consistent shifts toward younger predicted biological age across treatment groups (Zhavoronkov et al. 2026). Together, these studies highlight how molecular aging clocks can connect health trajectories with biological processes and provide quantitative tools for evaluating potential geroprotective effects of therapeutic interventions.

The particular value of aging clocks for convergent geroscience lies in their ability to connect population‐level exposures with measurable features of aging biology. A growing body of research demonstrates that social conditions lead to changes in biological aging clocks (Crimmins 2020). Early‐life adversity, including childhood poverty, abuse, and instability, is associated with accelerated epigenetic aging later in life, even in the absence of overt disease (Zannas 2019; Crimmins et al. 2024). Socioeconomic disadvantage across adulthood similarly predicts accelerated biological aging across epigenetic, proteomic, and metabolomic measures, reflecting sustained psychosocial stress, environmental exposure, limited healthcare access, and metabolic dysregulation (Crimmins et al. 2024; Harris et al. 2024). Recent transcriptomic approaches further extend these observations. For example, Transcriptomic Mortality‐risk Age (TraMA), an RNA‐seq‐based measure developed in the population‐based Health and Retirement Study, was associated with socioeconomic and behavioral characteristics as well as mortality, multimorbidity, cognitive function, and functional limitations, illustrating how transcriptomic aging measures can connect population‐level heterogeneity with molecular and health‐related dimensions of aging (Klopack et al. 2025). Chronic occupational strain, poor work‐life balance, and persistent psychological stress activate stress‐response pathways characterized by elevated cortisol, immune dysregulation, and chronic inflammation (Zannas 2019; The BIOS Consortium et al. 2019). Importantly, these associations are not limited to composite aging clocks but are also evident across individual biomarkers measured in population‐based studies, including inflammatory markers, endocrine signals, and metabolic indicators. Thus, biological aging clocks provide a quantifiable framework through which social and psychological environments can be measured at the molecular level even in population studies, complementing a broader set of biomarkers that capture these processes across different timescales and physiological systems.

In this sense, aging clocks exemplify convergence in action. They enable testing whether social interventions modify biological aging trajectories, allow stratification of populations by biological vulnerability rather than chronological age, and provide tools to evaluate gerotherapeutics within socially heterogeneous cohorts. The rapid evolution from methylation‐based clocks to multi‐omic and systems‐level frameworks underscores a broader transformation of geroscience toward integrative, population‐informed biology, demonstrating that social determinants are increasingly recognized as important contextual factors associated with the trajectory of human aging (Table 1).

One important consideration is that aging clocks should not be interpreted as direct mechanistic readouts of aging processes. Most clocks are optimized for predictive performance rather than mechanistic specificity, and many methylation features incorporated into clock models may function primarily as biomarkers rather than causal drivers. Recent approaches have begun to address this limitation by incorporating causal inference into clock development; for example, one study used Mendelian randomization to identify CpG sites with putative causal relationships to aging‐related traits and developed causality‐enriched clocks that distinguish potentially damaging from adaptive age‐associated methylation changes (Ying et al. 2024). Overall, the ability of aging clocks to reproducibly capture variation associated with social, environmental, and physiological exposures provides a valuable framework for studying how population‐level experiences become biologically embedded.

### 4.2 Beyond Aging Clocks: Additional Examples of Convergent Geroscience

Although biological aging clocks provide a particularly clear example of convergent geroscience, several other established areas of aging research illustrate how population and experimental biology approaches can inform one another. Telomere length provides another example of a biological measure that can bridge population and experimental biology research. Telomeres progressively shorten with repeated cell division in many somatic tissues, and critically short or dysfunctional telomeres can activate DNA damage responses, promote replicative senescence, and contribute to genomic instability, providing a mechanistic connection between telomere attrition and cellular aging (López‐Otín et al. 2023; Chakravarti et al. 2021). At the population level, shorter leukocyte telomere length has been associated with diverse social and environmental exposures, including early‐life adversity, psychosocial stress, socioeconomic disadvantage, and environmental stressors (Ridout et al. 2018; Pepper et al. 2018). Thus, unlike biomarkers developed primarily for age prediction, telomere biology illustrates how a molecular process with experimentally defined consequences can also be measured across human populations to investigate how lived exposures relate to biological aging. However, interpretation requires some caution as leukocyte telomere length varies substantially across individuals and cell populations, and estimates are influenced by tissue composition, measurement methodology, and other biological and technical factors (Montpetit et al. 2014). These limitations reinforce the value of integrating population observations with experimental approaches capable of testing the mechanisms underlying observed associations.

Conversely, population‐derived genetic observations illustrate how convergence can proceed in the opposite direction. The identification of *APOE* ε4 as a major genetic risk factor for Alzheimer's disease emerged from studies of human populations (Corder et al. 1993) and subsequently stimulated decades of experimental work investigating how ApoE influences disease biology. These studies have implicated ApoE in processes including lipid transport and metabolism, amyloid‐β aggregation and clearance, neuroinflammation, and neuronal vulnerability (Windham and Cohen 2024; Yamazaki et al. 2019; Raulin et al. 2022; Qian et al. 2022; Huang and Mahley 2014), transforming a population‐level genetic association into a broad program of mechanistic investigation. Importantly, *APOE* also illustrates why the exchange need not end with identification of a mechanism: the magnitude and consequences of *APOE*‐associated risk vary across human populations and environmental contexts, providing additional questions that can be returned to experimental systems for mechanistic investigation. Thus, population observations, whether genetic, social, or environmental, can identify biologically important variation, while experimental models provide the controlled systems needed to determine how that variation alters aging and disease processes.

### 4.3 Emerging Model Systems Informed by and Informing Population Research

The development of model organisms historically prioritized practical and experimental considerations, including low cost, short generation times, genetic tractability, and the ability to generate large, genetically controlled populations. Invertebrate model systems boast very low laboratory costs, as well as the ability to generate a large number of animals with virtually identical genomes. However, a growing class of model systems has recently emerged based on insights from social and behavioral population research. These models were chosen because their behavioral, social, or ecological niches allow for mechanistic studies that conventional laboratory species could not capture. These systems are particularly valuable for human biology because they allow experimental investigation of how social structure, behavior, and environment shape physiological processes relevant to human health and aging.

Socially specialized models provide one example of how experimental systems can be selected to interrogate population‐relevant exposures. Prairie voles ( *Microtus ochrogaster* ), for example, form long‐term pair bonds and exhibit social behaviors regulated by oxytocin‐ and vasopressin‐dependent neural circuits, providing an experimentally tractable system for investigating how social relationships influence physiology (Insel 2010; McGraw and Young 2010; Horie et al. 2019). Given strong population‐level associations between social relationships and health outcomes (House et al. 1988), such models may ultimately help define mechanisms linking social environments to aging.

Several additional model systems including the African turquoise killifish ( *Nothobranchius furzeri* ) and tardigrades emerged from environmental and ecological insights, and in turn offer great potential to understand human health. The African turquoise killifish originally arose as an environmental and evolutionary model to understand how growth in ephemeral seasonal pools required evolution of accelerated maturation and reproduction (Reichard and Polačik 2019). In the wild, African turquoise killifish live only a few months on average, as their source of water exists only during the wet season, followed by a dry season in which they must exist in a drought‐resistant diapause state (Polačik et al. 2018). This natural life cycle was then leveraged by aging biologists, who found that African turquoise killifish had a lifespan of approximately 4–6 months in captivity, making them the shortest‐lived vertebrate model system that can be bred in captivity (Hu and Brunet 2018), enabling longitudinal aging studies at a speed not possible in traditional vertebrate systems including mice and zebrafish. Since its introduction to geroscience, the African turquoise killifish model has transformed multiple areas of aging biology including brain aging (Vanhunsel et al. 2021; de Bakker and Valenzano 2023), immune aging (Le Bras 2022), and muscle aging (Ruparelia et al. 2024). In addition, its unique ability to enter into a suspended‐animation, non‐aging diapause state revealed striking mechanisms that could underlie aging resistance (Hu and Brunet 2018). Diapause engages conserved aging pathways such as reduced insulin/IGF signaling and mTOR activity, enhanced stress resistance, and maintenance of proteostasis, mechanisms that are also implicated in lifespan extension and healthy aging in humans, thus having the capacity to inform mechanistic understanding of how these pathways impact human health.

Tardigrades provide another example of an experimentally tractable system shaped by extreme environmental pressures. Their remarkable tolerance to desiccation, temperature extremes, and DNA damage is supported by specialized stress‐protective mechanisms, including tardigrade disordered proteins that stabilize proteins and cellular structures (Lim et al. 2024; Hesgrove and Boothby 2020; Boothby et al. 2017; Smythers et al. 2024). Expression of tardigrade stress‐resilience proteins can influence longevity in other experimental systems (Richaud et al. 2025), highlighting their potential for identifying conserved mechanisms of stress resistance. Such models may become particularly relevant as population studies increasingly associate environmental stressors, including extreme heat, with altered biological aging (Choi and Ailshire 2025).

Across these examples, behavioral, social, and ecological observations have served as entry points for the development of innovative laboratory models that reveal phenotypes not accessible in classical systems. These models, in turn, can be used to provide novel biological insights relevant to social and environmental factors that drive human aging. These emerging models expand the already powerful experimental toolkit of geroscience by enabling causal investigation of biological responses to social, behavioral, and environmental conditions that are difficult to model in traditional systems.

### 4.4 Utilization of Population Approaches to Heterogeneity to Improve Experiments Using Animal and Cell Models

Population research has long embraced that evaluation of heterogeneity, including genetic, environmental, and phenotypic variability, is necessary and informative to understanding human health. Such approaches are also common in evolutionary biology. In contrast, experimental biology typically seeks to control and reduce heterogeneity to better isolate specific mechanisms. However, recent shifts show that population approaches that explore and leverage heterogeneity can actually strengthen the relevance of model organism research for understanding human aging. Recognizing and studying variability not only improves experimental reproducibility but also helps align laboratory models with the heterogeneity observed in human populations. For example, even canonical model systems, such as *C. elegans* , immortalized cell lines, and laboratory mice, harbor previously underappreciated sources of biological variability.

The value of interrogating rather than eliminating heterogeneity is apparent even in *C. elegans* , a model historically prized for the ability to generate genetically identical populations. Despite this apparent uniformity, substantial differences in lifespan have been reported among N2 stocks across laboratories (Urban et al. 2021; Sterken et al. 2015), and genomic analyses have identified mutations acquired during laboratory domestication that can alter aging phenotypes (Zhao et al. 2019; Andersen et al. 2012). At the same time, studies exploiting natural variation across hundreds of wild *C. elegans* isolates and recombinant populations have identified genetic variants associated with lifespan, stress resilience, development, pathogen resistance, and drug responses (Snoek et al. 2019; Zhang et al. 2022). Together, these studies demonstrate how controlled mechanistic experiments and population‐level analysis of natural variation can complement one another, revealing aging mechanisms that may not be apparent within a single genetic background.

In mouse work, population approaches to variability have likewise reshaped experimental design for aging biology. While classic mechanistic work still relies heavily on inbred strains such as C57BL/6J mice to minimize variability, intervention studies now widely use genetically heterogeneous populations. As a leading example, the NIA's Interventions Testing Program (ITP) intentionally introduces genetic and environmental variables into their research. The ITP is a large‐scale, rigorously controlled initiative that evaluates promising lifespan and healthspan extending interventions, utilizing the genetically diverse UM‐HET3 mouse line and conducting parallel studies at three independent sites to ensure that any potential gerotherapeutic discoveries can withstand genetic and environmental diversity (Nadon et al. 2008). Importantly, the program systematically tests candidate gerotherapeutics, including compounds currently in use or under investigation in human medicine, to determine whether their effects on longevity are robust across genetically diverse populations.

In addition to the NIA initiative, the Diversity Outbred mouse derived from eight inbred founder strains and maintained through randomized breeding was recently developed to model the high genetic diversity of humans (Churchill et al. 2012). This population is now widely used in aging research, and a large meta‐analysis of lifespan data from 2444 mice across three independent studies identified multiple genetic loci associated with lifespan across dietary and pharmacological interventions (Mullis et al. 2025). This study found that these loci could explain over half of the estimated heritable variation in lifespan and highlights one of the first genetically diverse cohort studies in mice that mirror population‐based aging studies in humans. Together, these approaches demonstrate how incorporating heterogeneity into model systems can improve the translational relevance of preclinical findings, ensuring that candidate interventions are robust to the variability inherent in human populations.

Collectively, these examples illustrate how importing concepts from population science, including human health research and evolutionary biology, back into experimental biology can both contextualize and improve fundamental experiments. The use of standardized models such as N2 worms or C57BL/6J mice has been invaluable for reducing biological variability and enabling rigorous mechanistic discovery. At the same time, these models remain dynamic biological populations in which genetic drift, environmental history, and heterogeneous responses can emerge over time. Recognizing and characterizing these sources of variation can complement traditional controlled approaches by revealing how established mechanisms operate across different biological contexts and by identifying factors that contribute to variability in experimental outcomes. Ultimately, population‐level studies even in model systems can help better integrate fundamental biology studies into a more macroscopic level to identify geroscience interventions that can withstand the test of variability. In this way, population research is not only useful to understand human aging but can be used to refine experimental bench science, even at the fundamental level of model organisms.

### 4.5 Sex as a Biological Variable: From Population Insight to Mechanistic Geroscience

Recognition that males and females experience health and aging differently, informed by decades of public health and demographic research, provides a clear example of how population observations can inform experimental biology. One of the most striking observations is the persistent female survival advantage: women generally live longer than men, yet experience higher rates of some diseases, disability, and physical and cognitive decline later in life (Tran et al. 2021; Gronich et al. 2024; Phyo et al. 2024; Brown et al. 2022). This discordance demonstrates that increased lifespan does not necessarily reflect slower or healthier aging and suggests that males and females can follow distinct trajectories in the onset and progression of biological aging. Understanding the mechanisms underlying sex‐specific vulnerability and resilience has therefore become important for developing interventions that benefit both males and females.

Aging research, particularly studies using model organisms, has historically been overwhelmingly male dominated (Seydel 2021). Evidence from multiple analyses shows that males were often treated as the default biological model, based on the assumption that cyclical hormonal fluctuations in females would increase experimental variability (Beery and Zucker 2011; Ng and Hazrati 2022; Yoon et al. 2014; Carmody et al. 2022). This approach was further justified by the misconception that findings from males could be directly generalized to females (Woitowich et al. 2020). As a result, aging research produced male‐biased models. Due to mounting concern that these biases were undermining scientific rigor and translational relevance, the NIH implemented policies mandating inclusion of women in clinical research in 1993 (Clayton and Collins 2014), and in 2016 the NIA required explicit consideration of sex as a biological variable in the design, analysis, and reporting of preclinical research it funds (Carmody et al. 2022). These mandates formalized what population‐based research had long demonstrated: sex differences in aging are biologically meaningful and must be mechanistically understood. Although recent studies have documented improved inclusion of both sexes in preclinical aging research, meaningful sex‐specific analyses remain inconsistent (Carmody et al. 2022). Even when male and female animals are included, data are often pooled, sex is regressed out or treated solely as a covariate, and analyses proceed without accounting for potentially large sex effects (Seydel 2021; Beery and Zucker 2011; Woitowich et al. 2020; Will et al. 2017). These approaches implicitly assume linearity or equivalence between sexes, potentially obscuring distinct mechanisms and differential responses to aging interventions.

As sex‐inclusive approaches have expanded, accumulating evidence has revealed robust biological differences across multiple domains of aging. Biological sex shapes aging through both sex chromosome‐linked mechanisms and gonadal hormone‐dependent pathways, influencing metabolism, immune function, and inflammatory signaling. GWAS have identified sexually dimorphic genetic loci involved in fat distribution and metabolic regulation, with female‐biased effects on waist‐to‐hip ratio (Heid et al. 2010). A growing body of literature further demonstrates that biological sex shapes both innate and adaptive immunity in peripheral and central immune systems (Heid et al. 2010; Debarba et al. 2022; Osborne et al. 2018; Dunn et al. 2024; Della Peruta et al. 2023). The X chromosome is enriched for immune‐ and inflammation‐related genes, and studies using gonadectomized animal models indicate that gonadal hormones exert major regulatory roles across the lifespan (Debarba et al. 2022; Dunn et al. 2024). These findings provide mechanistic grounding for epidemiological observations and underscore why aging trajectories differ between males and females.

These biological differences also have direct implications for gerotherapeutic development. Candidate interventions targeting pathways implicated in aging can exhibit different efficacy or physiological effects between males and females, including sex‐specific responses to hormonal and metabolic interventions (Debarba et al. 2022). Thus, biological sex is not simply a source of experimental variability but an important modifier of aging mechanisms and therapeutic responsiveness. Mechanistically defining these differences can therefore improve both interpretation of preclinical studies and development of interventions that are effective across heterogeneous populations.

Importantly, biological sex represents only one component of sex‐ and gender‐associated heterogeneity. Population research also highlights the contribution of gendered social experiences, including discrimination and differential environmental or behavioral exposures, which may shape aging trajectories through distinct but interacting pathways (Krieger 2020). These social dimensions are not readily reproduced in traditional animal models, creating an important opportunity for population research to identify relevant exposures and for experimental approaches to investigate how those exposures become biologically embedded.

Viewed through the lens of convergent geroscience, sex differences therefore illustrate the value of iterative exchange between population and experimental biology research. Demographic and epidemiological studies identify divergent aging and disease trajectories, while experimental biology defines the chromosomal, hormonal, immune, and metabolic mechanisms underlying these patterns. These mechanistic discoveries can in turn refine population studies and inform sex‐specific therapeutic strategies. Thus, population observations can identify biologically important heterogeneity, while experimental systems provide the causal resolution needed to explain and ultimately intervene upon it.

## 5 Multidisciplinary Scientific Meetings as Forums for Integrative Geroscience

The expanding landscape of aging research, spanning molecular hallmarks of aging, social determinants of health, experimental model systems, and large‐scale population cohorts, underscores a central theme of this review: no single disciplinary framework is sufficient to fully explain the complexity and heterogeneity of human aging. Increasingly, efforts across the field have emphasized the value of integrating mechanistic biology with population‐level observations and human variability.

The Geroscience Los Angeles Meeting (GLAM) emerged in part from this broader recognition and was established as an interdisciplinary forum bringing together researchers across experimental biology, translational medicine, psychology, clinical research, and population science. The scientific scope of GLAM spans topics including stem cell aging, senescence biology, neurobiology of aging, translational geroscience, social hallmarks of aging, population health, and health disparities. More broadly, this shift toward integrative geroscience is increasingly reflected across the aging field. For example, the Gerontological Society of America (GSA), historically centered on social and population aging research, has expanded engagement with biological geroscience, while the American Aging Association (AGE), traditionally focused on mechanistic biology of aging, has increasingly incorporated translational, clinical, and population health perspectives. Together, these developments reflect growing recognition that understanding and intervening in aging will benefit from approaches that integrate mechanistic, clinical, and population‐level perspectives. Continued support for interdisciplinary and integrative aging research across funding, publication, and scientific meeting structures may help accelerate progress in geroscience.

## 6 Why This Matters Now: Forward Thinking

Global increases in lifespan accompanied by rising chronic disease burden create growing urgency to translate geroscience discoveries into scalable interventions that preserve health and function. Achieving this goal will require integrating the mechanistic foundation established by experimental biology with the genetic, environmental, behavioral, and social heterogeneity revealed by population research.

We envision convergent geroscience as a bidirectional and iterative process. In one direction, mechanisms and interventions identified through experimental biology are evaluated across heterogeneous human populations to establish their relevance across genetic, environmental, behavioral, and social contexts and improve translation. In reverse, population research identifies exposures, phenotypes, or aging trajectories that are subsequently modeled in experimentally tractable systems to establish causality, define underlying mechanisms, and identify potential interventions. These directions are complementary rather than discrete and discoveries can move repeatedly between experimental systems and human populations as mechanisms, contexts, and interventions are progressively refined.

Operationalizing these bidirectional approaches will require coordinated changes across experimental biology and population research. Experimental systems should retain the controlled designs essential for mechanistic discovery while incorporating complementary studies that test established mechanisms across defined sources of genetic, environmental, and physiological heterogeneity. Population cohorts can increasingly integrate molecular biomarkers with detailed social and environmental measures to examine how exposures relate to aging biology longitudinally. Hybrid designs linking human observational data with experimentally tractable systems, or embedding mechanistic substudies within large cohorts, can further strengthen causal inference and identify actionable targets.

Existing translational pipelines can similarly be strengthened by incorporating heterogeneity earlier in the evaluation of geroscience interventions, including stratification by biological, environmental, and social risk profiles and testing interventions across diverse populations earlier in development. Shared platforms integrating molecular, clinical, and population data could further facilitate bidirectional discovery, while interdisciplinary training and funding mechanisms can provide the infrastructure needed to sustain these approaches.

Ultimately, convergent geroscience will require research structures that facilitate movement between mechanistic and population science, including collaborative funding mechanisms and review panels with expertise spanning these domains. The goal is not to replace the approaches that built modern geroscience, but to connect them more deliberately, using experimental biology to establish mechanism and population research to define context, then iterating between the two to identify, validate, and translate interventions that meaningfully extend healthspan across populations.

## Author Contributions

N.D. and R.H.‐S. wrote the manuscript. E.H.‐L. and J.A.A. assisted in preparation of population health and social science sections. V.A.A. and B.A.B. wrote the sex differences section. G.G. prepared all figures. E.M.C. and S.P.C. assisted in editing and factchecking of the manuscript. All authors revised and approved the manuscript.

## Funding

This work was supported by the National Institute on Aging (R01AG079806, R00AG075317, P30 AG017625, R01 AG076433), National Institute of General Medical Sciences (R35 GM142395), Hevolution Foundation (HF‐GRO‐23‐1199072‐28), Simons Foundation (SF811217).

## Conflicts of Interest

The authors declare no conflicts of interest.

## Data Availability

Data sharing not applicable to this article as no datasets were generated or analysed during the current study.
